# Nietzsche

**Published:** 1914-12

**Authors:** 


### Reviews of Books.

**Nietzsche.** By J. M. KENNEDY. Pp. 192. London: T. Werner Laurie, Ltd. 1914. 1s. net.

Nietzsche was a German, a bit of a genius, and a crank, who finished up, appropriately enough, as a lunatic. The German nation, whose religion he described as at best a lifeless Lutheranism, and at worst a pernicious hypocrisy, is believed in England, just at present (1914), to have abandoned that religion at his request, and taken his works as gospel and *vade mecum*. This is one of those beliefs that flourish because of their rank absurdity. Nietzsche was disgruntled, hipped and gravelled with his German surroundings and everything German. He gave his fellow-countrymen the greatest scourging any nation has received since the days of the Prophet Jeremiah. But his philosophy has no coherence, and a German who said,

"I believe in Nietzsche," might as well say, "I believe in Edward Lear's collected Nonsense Works." Possibly the Germans are being persuaded that the British Empire believes the teachings of G. B. Shaw, in which case they are making the same mistake about Shaw that we make about Nietzsche. "Develop yourself and don't be a slave," is the most useful part of his advice to the world. He wanted to see a Europe imbued with this doctrine, and scraped clean from the barnacles of culture in which Germany is so prolific. He could not predict where the lead would come from—it would need men and supermen—but certainly, of all nations, it would never come from the Germans, men who reached the limit of pack-hound servility.

The disastrous consequences of too much learning were known to the "most noble Festus," and even earlier observers. That was before science had begun to unpack her stores, and while yet history was a happy field for flowers of the imagination. It was not too much learning that drove Nietzsche into an asylum—he has some penetrating shafts to fling at learned pedantry—but too much brooding. No doubt the portion of our planet labelled German Empire is full of people "gey ill to live with," but Nietzsche's real quarrel was with the planet at large, and, in the last analysis, with himself. As a rule, if a man cannot be comfortable where he is he cannot be comfortable anywhere.

Nietzsche got hold of certain principles, "survival of the fittest" in the main, which were in the air at the time, and worked them to the bitter end. Perhaps Darwin came near to surprising one of Nature's secrets, but the German wanted to go farther, and imagined man could use this secret in his own way for his own purposes, leaving Nature to echo the lament, "Othello's occupation 's gone." Folk-lore is strewn with parables hinting at the deadly danger of this attempt—the Sphinx, Bluebeard's Cupboard, and the Tree of Knowledge. "Glissez, mortels, n'appuyez pas," should be the motto of a philosopher who wishes to retain his digestion, sanity and content. Even for the man of genius a bath in the commonplace, among commonplace people, including so-called fools, is a wholesome antiseptic and tonic. Truth is so many-sided, that any single side of it, especially one's own, is bound to be false. Nietzsche exposed the tender parts of his skin, perversely, to the prickles of life; more sensible people delegate the duty of encountering prickles to the indurated portions of their epidermiz.

Nietzsche exhibits, however, the characteristics which Germans have copied with such assiduity and success, that they permeate apparently the whole nation—swelled head, hardening of the heart, and softening of the brain. The frog tries to become the ox, and declares a world war; or, even more pathetic, tries to become a butterfly and coin epigrams, at which game the French easily fly over him. Confronted by a rainbow, and incapable of rejoicing in its beauty, the super-German will chop it up and reduce it to hideousness. It is a queer taste and trade, as if a sausage maker should boast of converting good meat into a cylinder of superlative nauseabundity.

Mr. Kennedy's selection is judiciously compiled. If the reader is immune from attacks of philosophico-melancholia he can turn to Nietzsche as to a quarry of arresting thoughts, some of which come as near to being epigrams as anything of German utterance. Without any delicacy, humour or wit, his sentences have relief. He stands at work like a wild stone-mason (the cover of the book has a good portrait of him, with hair and features of palæolithic man, and eyes of a wolf trapped in a cave). Such an artist is bound to attract a crowd as soon as the cry is passed along: "Hi, boys, come and see him make the chips fly!" He is nothing like as original as he fiercely claims to be. Thus the theory of the recurrent world-cycle is older than Virgil, but he is daring and destructive, and readers of this shilling book, if they did their borrowings with discretion, would be able to pose as regenerators of society, to the great discomfiture of the common herd.

